# Head and Neck Manifestations of Tularemia in Tyrol (Austria): A Case Series

**DOI:** 10.3390/diagnostics15091138

**Published:** 2025-04-29

**Authors:** Roland Hartl, Matthias Santer, Wegene Borena, Charles Schmit, Hannes Thomas Fischer, Daniel Dejaco, Benedikt Gabriel Hofauer, Teresa Bernadette Steinbichler

**Affiliations:** 1 Department of Otorhinolaryngology-Head and Neck Surgery, Medical University of Innsbruck, Anichstr. 35, 6020 Innsbruck, Austria; roland.hartl@tirol-kliniken.at (R.H.);; 2 Institute of Virology, Department of Hygiene, Microbiology, Social Medicine, Medical University of Innsbruck, Peter-Mayr-Str. 4B, 6020 Innsbruck, Austria

**Keywords:** tularemia, rabbit fever, zoonosis, cervical lymphadenopathy, lymph node abscess, rare disease

## Abstract

**Background:** Tularemia is a rare zoonosis caused by the bacterium *Francisella tularensis*. In the head and neck region, it can manifest as cervical lymphadenopathy. Despite intensive therapy with various antibiotics, there is often a prolonged medical course. **Methods:** In this paper, all documented cases of tularemia in the head and neck region at the Medical University of Innsbruck (Austria) are analyzed and the results compared with the literature. A retrospective analysis of all patients diagnosed with tularemia at the Department of Otorhinolaryngology-Head and Neck Surgery, Medical University of Innsbruck (Austria), was performed. Tularemia was diagnosed using a serologic agglutination antibody test. **Results:** Thirteen patients with tularemia presented at the Department of Otorhinolaryngology-Head and Neck Surgery, Medical University of Innsbruck (Austria), between 2010 and 2024. In 10 patients (10/13; 77%), animal contact or an insect bite was the suspected cause. The mean time from the onset of the first symptoms to diagnosis was 36 ± 15 days. The therapy took a mean of 5 ± 2 months until the last follow-up. On average, the patients were treated with 4 ± 1 different antibiotics. The median duration of hospital stay was 13 days (range: 0–36). In addition, a median of 9 (range: 2–20) further outpatient check-ups with several neck ultrasounds were carried out. Also, 10 patients (10/13; 77%) received a diagnostic and/or therapeutic surgical intervention. **Conclusions:** Tularemia is a rare infectious disease with a prolonged diagnostic and therapeutic course. Screening for tularemia should be performed in cases of cervical lymphadenopathy, especially if empirical antibiotic treatment has been ineffective or if there is a specific medical history.

## 1. Introduction

George McCoy, M.D., discovered the pathogen Franciscella tularensis in 1911 [1]. He named it after Tulare County in California, USA. In 1919, Edward Francis, M.D. (surgeon), determined that an illness known as deerfly fever was an infection caused by the bacterium tularense and gave it the name tularemia [2,3,4]. In Europe, tularemia-like symptoms have been reported since the late 19th century. During the 20th century, Austria, France, Hungary, Bulgaria, and Germany experienced large outbreaks with several hundred cases of tularemia due to low hygienic conditions during wartime and increased agricultural activity [5].

Tularemia is a zoonotic disease transmitted from animals to humans. Up to 250 different animal species, including mammals, birds, fish, and arthropods, serve as vectors for the disease [6,7]. Pets such as rodents, cats, and rabbits are also involved in the spread of the disease [8]. Its transmission to humans occurs through the consumption of contaminated food and water, via the skin through bites from infected insects or animals, and through the inhalation of contaminated dirt [7,9,10]. So far, there has been no evidence of human-to-human transmission [11].

Different variants of tularemia are classified on the basis of their clinical appearance [9]. According to the WHO guidelines from 2007, the ulceroglandular, glandular, oropharyngeal, oculoglandular, respiratory, and typhoid forms can be distinguished [6,12]. The clinical symptoms depend largely on the route by which the bacterium enters the body [13]. The oropharyngeal form prevails in countries where precarious living conditions encourage the consumption of contaminated water and food, like in Turkey, Bulgaria, and Kosovo [14,15]. The glandular and the ulceroglandular forms of tularemia are the most common clinical variants of the disease in the United States of America and Europe [6,14]. They cover more than 95% of the reported cases [11].

Due to climate change, the number of people suffering from tularemia is expected to increase in the coming decades [16]. Eleven to forty-five percent of people with tularemia have symptoms or signs on their head and neck. For this reason, otolaryngologists should also consider tularemia as a differential diagnosis for lymph node swelling in order to avoid delayed diagnosis and treatment [10].

In this case series, we analyzed all patients who presented with tularemia at the Department of Otorhinolaryngology-Head and Neck Surgery, Medical University of Innsbruck (Austria), between 2010 and 2024.

## 2. Materials and Methods

A retrospective, pseudoanalytical data analysis was performed at the Department of Otorhinolaryngology-Head and Neck Surgery, Medical University of Innsbruck (Austria), between 2010 and 2024. Data were collected through a hospital-wide search for tularemia ICD code (A21.x) and the extraction of data from positive serological tularemia tests by the Department of Hygiene, Microbiology, and Public Health. Tularemia was diagnosed via serological test using microagglutination. The WHO guidelines suggest a cut-off antibody titer of ≥160 IgM for the microagglutination test [17,18]. Various patient and disease parameters were analyzed. These parameters included demographic data such as gender, age at diagnosis, profession, and ASA score (American Society of Anesthesiologists score), serving as a simple tool for assessing overall health status [19,20]. The data were also used to calculate the duration of symptoms until diagnosis and the total time until the end of medical care. The main symptoms and the laboratory results at the time of diagnosis were recorded. Moreover, we analyzed surgical interventions, antibiotic therapies, the duration of inpatient stays, and additional outpatient check-ups. Numeric data are presented as means ± standard deviations unless indicated otherwise. Data analysis was performed with SPSS-22 (IBM Corporation, Armonk, NY, USA).

### Ethical Considerations

This study was approved by the review board of the Medical University of Innsbruck, Austria (1395/2024; Date: 27 January 2025). All procedures conducted in this study involving human participants were in accordance with the ethical standards of the institutional review board and with the Helsinki declaration (1964) and its later amendments or comparable ethical standards.

## 3. Results

**Figure 1:**
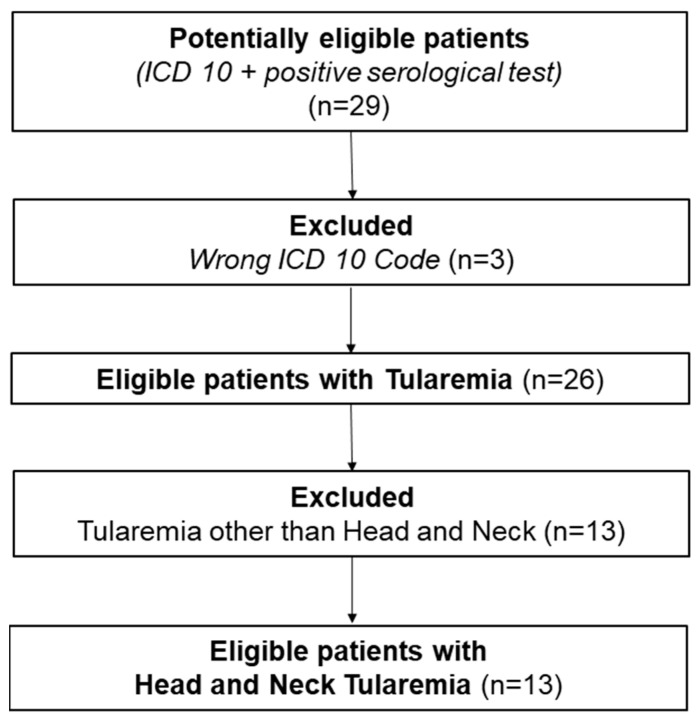
Collection of data: On the basis of a positive serological finding or a matching ICD code, 29 patients were identified. Of these, 3 patients were incorrectly coded. A further 13 patients had tularemia outside the head and neck region. Thus, 13 cases could be included in the analysis.Of these 13 patients, 3 were male (3/13; 23%) and 10 were female (10/13; 77%). The median age was 59 years (range: 1–75), as summarized in Table 1 along with further clinical and demographic data.

Between 2010 and 2024, 26 patients with tularemia were diagnosed and treated at the Medical University of Innsbruck (Austria). The data collection process is shown in Figure 1. Exactly half of them (13/26; 50%) had tularemia in the head and neck area, with the leading symptom being cervical lymphadenopathy.

Many of the patients (10/13, 77%) had a normal C-reactive protein (CRP < 1 mg/dL) and normal leucocytes (12/13, 92%). In 5 cases (5/13; 38%), the diagnostic serology showed a positive cross-reaction to the Bartonella henselae IgG-titer at a low level. In two cases, a polymerase chain reaction (PCR) analysis was performed, which showed the Francesiella tularensis subspecies holarctica (type B strains). The laboratory chemical data are summarized in Table 2.

In total, 10 of the 13 patients (10/13, 77%) probably became infected through animal contact or insect bites. A predisposing profession was reported in 4 of the 13 cases (31%). More than half of the tularemia patients (7/13, 54%) initially presented with swollen and/or inflamed palatine tonsils. Some (5/13, 38%) stated that they had a sore throat. Patients also reported other general, flu-like symptoms like stomach pain or initial diarrhea (2/13), weight loss (2/13), body pain (4/13), tiredness (5/13), and fever (8/13). In 10 patients (10/13, 77%), the first symptoms appeared in the summer or autumn months. The clinical data are further summarized in Table 3.

The mean time from onset of symptoms to diagnosis was 36 ± 15 days. In 4 cases (4/13; 31%), the initial treatment was carried out with amoxicillin/clavulanic acid. On average, 4 ± 1 different antibiotic therapies were prescribed per patient. Doxycycline and ciprofloxacin were prescribed as antibiotic therapy in 11 out of 13 cases (85%). The median duration of inpatient treatment of the patients was 13 days (range: 0–36). They each had a median of 9 outpatient check-ups (range: 2–20).

Due to the unclear lymphadenopathy, a diagnostic core needle biopsy was performed in 4 cases (4/13, 31%). Surgical abscess drainage was performed in 4 (4/13, 31%), and cervical lymph node extirpation was performed in 31% (4/13) of the patients. Additionally, 2 patients (2/13, 15%) received two surgical interventions. In both cases, the lymph nodes were subsequently surgically removed. In one case, this was necessary due to persistent painful swelling of the lymph nodes despite consistent conservative treatment. In the second case, this was carried out for further diagnostic clarification. Only 23% (3/13) had no diagnostic or therapeutic surgical intervention (Table 4). The therapeutic data are further summarized in Table 4. Despite prolonged antibiotic treatment, the median time to completion of medical monitoring was 5 months (range: 2–12). Therapeutic data are summarized in Table 3. The individual course of the disease are added in the Appendix (Table A1)

## 4. Discussion

Tularemia is a very contagious bacterial infection caused by the Gram-negative, pleomorphic, and strictly aerobic bacterium *Francisella tularensis* [9,21,22]. The bacterium Franscicella tularensis can survive for several weeks in water or moist soil. It is inactivated by heat treatment or through the local application of various solutions such as high-percentage ethanol [9,23]. Due to its high virulence and its ability to spread via contaminated aerosol droplets, the bacterium Franscicella tularensis is classified as a potential pathogen for bioterrorism [11,24]. There are several names for tularemia worldwide, such as rabbit, hare, deerfly, or lemming fever [25]. Although tularemia is particularly widespread in the Northern Hemisphere, it is rarely diagnosed in Central Europe [21]. Around 800 cases are registered in Europe every year [24]. No vaccine has yet been approved [11,24]. In Austria, the incidence is estimated at up to 0.2 per 100,000 inhabitants. Direct contact with contaminated animals and tick bites is the predominant mode of transmission. Well-known endemic areas are Lower Austria, Burgenland, Vienna, Styria, and Upper Austria [15]. A nationwide cross-sectional serological analysis among healthy adults in Austria showed that 0.5% of the population had detectable antibodies against *Francisella tularensis* [11]. The disease can be asymptomatic, but it can also lead to sepsis and, if not handled properly, death [10]. The actual ratio of symptomatic to asymptomatic cases of tularemia is not known [13]. Lifelong immunity after infection is assumed, though reinfections have been described in the literature [24]. We detected 13 patients with tularemia presenting in the head and neck area in 13 years; see Figure 1.

The bacterium is a demanding organism that grows poorly on standard culture media. Therefore, it is difficult to cultivate and isolate [10]. The diagnostic gold standard is a serological test [17]. The microagglutination test, the indirect immunofluorescence test, and the enzyme-linked immunosorbent assay (ELISA) test are currently the most commonly used methods. ELISA tests can detect specific antibodies within two weeks of disease assessment, compared to the two to three weeks required for the microagglutination and indirect immunofluorescence tests. The long-term persistence of anti-*Francisella tularensis* antibodies in patients with previous tularemia infection compromises the diagnostic specificity of all these tests [18]. Cross-reacting antibodies have also been described (especially with Brucella and Yersinia species) but usually at a low level [18]. We were able to see cross-positivity to Bartonella henselae in 38% of the patients. The inflammatory parameters were mostly normal at the time of diagnosis; see Table 2.

The genus Francisella includes seven species. One of them is *Francisella tularensis*, which basically has four subspecies [15]: *Francisella tularensis* subspecies tularensis (type A strains), *Francisella tularensis* subspecies holarctica (type B strains), *Francisella tularensis* subspecies mediasiatica, and *Francisella tularensis* subspecies novicida. Type A and type B are considered to be the primary causes of the disease in humans and animals. Type A is common in North America, while type B is found throughout the Northern Hemisphere, with a higher prevalence in Asia and Europe [5]. Accordingly, a PCR analysis was performed in 2 of the 13 cases, and *Francisella tularensis* holarctica (type B strains) was identified.

The recommended clinically effective antimicrobial agents of choice are aminoglycosides or fluoroquinolones for 10 to 14 days, or tetracyclines for 14 to 21 days. No resistance has been described with this therapy. In severe cases, aminoglycosides are recommended due to their high cure and minimal relapse rates [11].

The ultrasound examination we carried out showed that some of the lymph nodes were significantly enlarged and inhomogeneous. In some cases, the lymph nodes appeared fluid; in others, the image was characterized by a capsule-like growth. This appearance indicated inflammatory changes in the lymph nodes. Computed tomography showed the affected lymph nodes to be centrally hypodense, inflammatory, melted, and necrotic with a contrast-absorbing wall and no signs of invasion. Histological examination revealed necrotizing granulomatous lymphadenitis.

In the European Union, tularemia appears to have a seasonal pattern, with most cases occurring during the summer and autumn months, due to increased exposure to infected vectors and animals. Only the oropharyngeal form is more common during the autumn, winter, and spring months due to exposure to contaminated nutrients. However, when compared across countries, the seasonality pattern appears to be very different. For example, in Germany, most cases are reported during the winter months [5]. Most of the patients we discovered were infected in late summer and fall.

The clinical presentations are mainly related to the bacterium’s entry point, the virulence of the *Francisella tularensis* subspecies, the host’s immune status, and the inoculated bacterial dose [5,10]. The incubation period for tularemia is approximately three to five days. However, periods between one and twenty-one days have been described [10].

Tularemia starts with nonspecific generalized symptoms such as high fever, chills, myalgia, gastrointestinal complaints, and swollen lymph nodes [6,9]. From the swollen lymph nodes, the bacterium can spread throughout the rest of the body. However, this spreading phase is temporary and mainly occurs early in the infection process [25]. The diagnosis and treatment of tularemia are often delayed and difficult due to various nonspecific, flu-like symptoms with swelling of the lymph nodes [10,17]. In general, complications such as rashes, soft tissue abscesses, otitis media, meningitis, and brain abscesses have been reported [15]. Suppuration, pneumonia, meningitis, and a prolonged recovery time are also possible [9]. In our study, it took an average of 36 days from symptom onset for a diagnosis to be made. Moreover, it took an average of 4 months to recover after diagnosis.

The epidemiology of tularemia is diverse. Occupations such as farmers, medical workers, and veterinarians have an increased risk of infection [22]. In addition, the rural population that engages in outdoor activities such as hunting and fishing, or people who have close contact with pets, are more affected. These are therefore considered risk factors for tularemia infection [5]. In Austria, direct contact with contaminated animals and tick bites is the predominant mode of transmission [15]. Accordingly, some patients from our study group were gardeners, farmers, or hunters. More than 50% of the patients we detected were exposed at work or during sports activities. The main route of transmission observed in our patient population was through animals or insects.

In general, men are more frequently infected [5]. This is primarily due to direct contact with wild animals during the hunting season. However, infections are also common in children and women [15]. Around three-quarters of our patients were women, and 2 out of 13 patients were under 14 years old (Table 1).

In the ulceroglandular form of tularemia, a small local skin lesion, such as a scratch, cut, or insect bite, serves as an entry point [5]. This form combines a persistent, festering skin ulcer with localized lymphadenopathy in the corresponding skin area [15]. The glandular form is similar to the ulceroglandular form except for the missing primary skin lesion. The pneumonic form arises when the bacterium is inhaled [5]. Patients with this form develop pneumonia with symptoms such as shortness of breath, coughing, and painful breathing. Chest X-rays show infiltrates and hilar lymphadenopathy [9]. The ingestion of contaminated food or water can result in the oropharyngeal form of tularemia [5]. Symptoms include sore throat, signs such as pharyngitis, tonsillitis, enoral blisters, stomatitis, and swollen, painful lymph nodes [9,15,17]. In the oculoglandular form, the conjunctiva of the eyes serves as the entry point. Most of the time, the bacteria are unintentionally introduced into the eyes digitally. Characteristics of this form are ulcerations and nodules on the conjunctiva. Without therapy, the bacteria can spread to the surrounding lymph nodes, leading to lymph node swelling [25].

Typhoid tularemia is a systemic disease with neurological symptoms without a detectable bacterial entry point and without the local manifestations seen in other forms of the disease. It presents general, nonspecific symptoms such as fever, asthenia, headache, myalgia, and neurological symptoms, such as confusion, stupor, and behavioral changes that mimic typhoid symptoms [15,26,27]. This form often has a more severe course of the disease. Cases lacking signs of local physical findings are categorized as typhoid tularemia [28]. We have seen cases of ulceroglandular, glandular, and oropharyngeal forms of the disease. In some patients, the route of infection was documented as an insect bite. However, it is sometimes difficult to make an exact classification.

**Figure 2:**
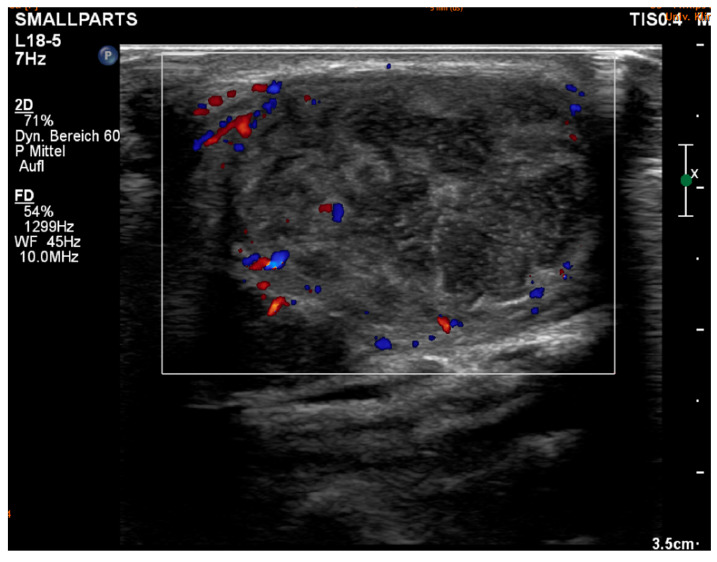
Ultrasound shows enlarged, inhomogeneous lymph nodes. In some cases, the lymph nodes appear liquid; in others, there is extracapsular overgrowth. This appearance suggests an inflammatory altered lymph node in the context of tularemia.

**Figure 3:**
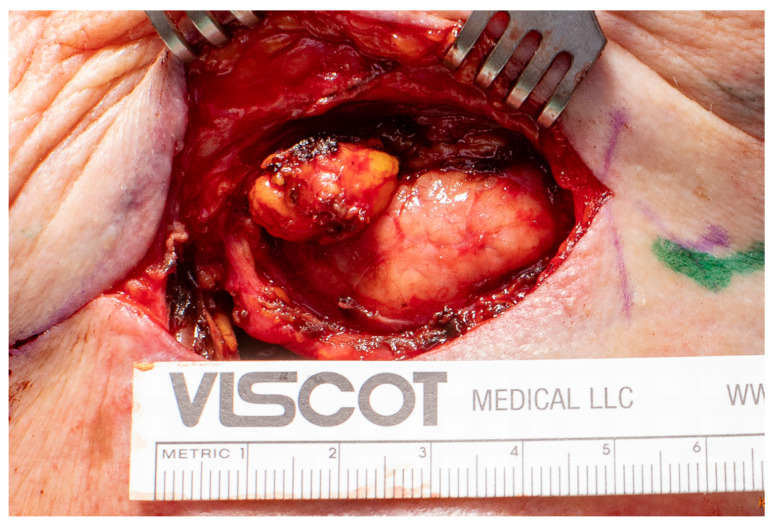
Intraoperative image of lymph node removal due to tularemia.

The diagnosis of tularemia is based on clinical examination, imaging techniques, and serological tests (Figure 2) an in rare cases after lymph node removal (Figure 3). Physicians should consider this disease a differential diagnosis in cases of unexplained lymphadenopathy that do not respond to treatment with beta-lactam antibiotics, especially if there are existing risk factors such as a predisposing occupation [10,24]. The disease can be transmitted to humans unnoticed, through the bite of an infected insect or tick and also via contact with the blood or internal organs of an infected animal. This means that market workers, cooks, hunters, and laboratory workers are also particularly at risk [29].

As otorhinolaryngologists, we focus on evaluating unclear, therapy-resistant cervical lymphadenopathy as the main symptom. Additionally, we have observed various accompanying symptoms such as abdominal pain, sore throat, aching limbs, fever, fatigue, weight loss, and diarrhea (Table 3).

With ongoing global warming, the potential area for the spread and the number of cases in Austria may increase significantly in the next few decades [30]. Although the mortality rate has fallen significantly in the era of antibiotics, tularemia is still considered a deadly disease that often requires escalating treatment with surgery or multiple antibiotics [10]. It is a potentially fatal illness and should not be underestimated [25].

## 5. Conclusions

Tularemia is a rare zoonosis caused by the bacterium *Francisella tularensis*, often manifesting as cervical lymphadenopathy. Patients with unexplained cervical lymphadenopathy should be screened for tularemia, particularly if empirical antibiotic therapy has proven ineffective or if there is a relevant medical or occupational history. Despite intensive treatment with various antibiotics, tularemia often follows a prolonged course.

## Figures and Tables

**Table 1:** Clinical characteristics of the 13 patients.

Sex	Male	3 (23%)
	Female	10 (77%)
Age	Median, range: 1–75	59 (years)
ASA Class	1	5 (38%)
	2	5 (38%)
	3	3 (23%)
Profession	Gardener/Farmer/Huntsman	4 (31%)
	Infant/Student	3 (23%)
	Service sector	2 (15%)
	Unknown	2 (15%)
	Retired	2 (15%)

**Table 2:** Laboratory chemical data: The inflammation laboratory at the time of diagnosis (CRP, leucocytes), the positive agglutination titer, and possible cross-reactions were documented. An assignment to a form of tularemia was also determined.

CRP	<1	10 (77%)
(in mg/dL)	1–3	1 (8%)
	3–5	2 (15%)
Leukocytes	Normal	12 (92%)
	Elevated	1 (8%)
Sub. Franciscella	Ulceroglandular Glandular Oropharyngeal or Glandular	3 (23%) 7 (54%) 3 (23%)
Franc. Tularensis Agglutinations titer (Normal value: <1:40)	1:160 1:320 1:640 1:1280 1:2560	2 (15%) 8 (62%) 1 (8%) 1 (8%) 1 (8%)
Bartonella henslae Laboratory chemical cross-reaction IgG/IgM	pos neg non	5 (38%) 7 (54%) 1 (8%)

**Table 3:** Clinical and therapeutic data: The patient’s clinical condition and the most important symptoms were recorded from their medical history. The route of infection was also specified. The various conservative and surgical treatment methods were analyzed. (*) = multiple entries per case are possible.

Tonsils swollen/tonsillitis	Yes	7 (54%)
	No	6 (46%)
Mode of transmission	Hare	4 (31%)
	Animal contact other than hares	6 (46%)
	Plants	1 (8%)
	Unknown	2 (15%)
Leading symptoms (*)	Cervical lymphadenopathy	13 (100%)
	Lymph node pain	11 (85%)
	Lymph node abscess	8 (62%)
	Stomach pain	1 (8%)
	Tiredness	5 (38%)
	Fever	8 (62%)
	Sore throat	5 (38%)
	Weight loss	2 (15%)
	Body pain	4 (31%)
	Initial diarrhea	1 (8%)
Season at symptom onset	Spring Summer Autumn Winter	2 (15%) 5 (38%) 5 (38%) 1 (8%)
Time from onset of symptoms to diagnosis	Median, range: 13–70	36 (days)
Duration of medical care	Median, range: 2–12	5 (months)

**Table 4:** Therapeutic data: The various conservative and surgical treatment methods were analyzed. (*) = multiple entries per case are possible.

Antibiotics prescribed for therapy (*)	Doxycycline	11 (85%)
	Clindamycin	4 (31%)
	Ciprofloxacin	11 (85%)
	Levofloxacin	2 (15%)
	Cefuroxime	6 (46%)
	Cefalexin	1 (8%)
	Amoxicillin/Clavulacin acid	5 (38%)
	Piperacillin/Tazobactam (i.v.)	2 (15%)
	Rifampicin	1 (8%)
	Amikacin (i.v.)	3 (23%)
	Gentamcin (i.v.)	4 (31%)
Number of given antibiotics	Mean	4
Total stationary duration	Mean Median, range: 0–36	13 (days) 13 (days)
Total ambulant checks	Mean Median, range: 2–20	10 9
Surgical intervention	Yes	9 (69%)
	No	4 (31%)
Surgical intervention (*)	Abscess cleavage	4 (31%)
	LN-Extirpation	4 (31%)
	Panendoscopy	3 (23%)
	CNB	4 (31%)
Secreting abscess wound/ Wound healing disorder	Yes No	5 (38%) 8 (62%)

## Data Availability

The data presented in this study are available upon request from the corresponding author.

## Appendix A

**Table A1:** Individual course of the disease.

Gender	Age at Diagnosis (in Years)	Profession	ASA Class ^1^	Mode of Transmission	Tonsils Swollen/Tonsillitis	Sub. Franciscella	Time from Onset of Symptoms to Diagnosis (in Days)	Leading Symptoms ^2^	Antibiotics ^3^	Duration of Symptoms Total (in Months)
Female	54	Gardener/Farmer/Huntsman	3	Unknown	Yes	glandular	23	1, 2, 3, 4, 5, 6, 7, 8, 9	1, 2, 3, 4, 10, 11	4
Female	61	Unknown	1	Animal contact other than hare	Yes	oropharyngeal or glandular	34	1, 2, 3, 6, 9	2, 7, 8, 3	4
Female	68	Gardener/Farmer/Huntsman	3	Unknown	Yes	oropharyngeal or glandular	40	1, 2, 3, 9	7, 1, 3	6
Male	61	Gardener/Farmer/Huntsman	2	Hare	No	glandular	39	1, 3, 5, 6, 9	10, 3, 1	6
Female	59	Service sector	2	Hare	Yes	ulceroglandular	42	1, 2, 3, 5, 6, 8	7, 5, 3, 10, 1	5
Female	18	Infant/Student	1	Animal contact other than hare	No	glandular	13	1, 2, 5	5, 1	2
Female	43	Service sector	1	Plants	No	glandular	70	1, 2, 3, 5, 6	1, 3	12
Female	74	Retired	2	Animal contact other than hare	Yes	ulceroglandular	25	1, 2, 3	2, 8, 1, 10, 3	5
Female	12	Infant/Student	1	Animal contact other than hare	No	glandular	50	1, 2, 6, 7, 10	7, 5, 1, 3	5
Male	70	Retired	3	Animal contact other than hare	Yes	ulceroglandular	49	1, 2, 3, 7	7, 5, 1	4
Female	75	Gardener/Farmer/Huntsman	2	Hare	No	glandular	23	1, 2, 7	5, 3, 4, 1, 9, 10, 4	5
Female	35	Unknown	2	Animal contact other than hare	No	glandular	23	1, 6	6, 1, 3, 11	7
Male	1	Infant/Student	1	Hare	Yes	oropharyngeal or glandular	31	1, 2, 3, 6, 7	5, 2, 11, 3	3

^1^. American Society of Anesthesiologists score (ASA). ^2^. 1 = lymphadenopathy; 2 = lymph node pain; 3 = lymph node abscess; 4 = stomach pain; 5 = tiredness, fatigue; 6 = fever; 7 = sore throat; 8 = weight loss; 9 = body ache; 10 = initial diarrhea. ^3^. 1 = doxycycline, 2 = clindamycin, 3 = ciprofloxacin, 4 = levofloxacin, 5 = cefuroxime, 6 = cefalexin, 7 = amoxicillin/clavulacin acid, 8 = piperacillin/tazobactam (i.v.), 9 = rifampicin, 10 = amikacin (i.v.), 11 = gentamcin (i.v.).
